# Plan quality comparison for cervical carcinoma treated with Halcyon and Trilogy intensity-modulated radiotherapy

**DOI:** 10.7150/jca.32500

**Published:** 2019-10-15

**Authors:** Chengqiang Li, Jinhu Chen, Jian Zhu, Guanzhong Gong, Cheng Tao, Zhenjiang Li, Jie Lu, Yong Yin

**Affiliations:** Department of Radiation Oncology Physics, Shandong Cancer Hospital and Institute, Shandong First Medical University and Shandong Academy of Medical Sciences, Jinan 250117, China.

**Keywords:** Intensity-modulated radiotherapy, Flattening filter free, Dual-layer MLC, Segment area

## Abstract

**Purpose:** Varian Halcyon is a novel machine with dual-layer leaves, single flattening filter free (FFF) energy and an enclosed bore. The purpose of this study was to compare the differences in dosimetry and plan parameters of intensity-modulated radiation therapy (IMRT) plans between the Halcyon and Trilogy accelerators.

**Methods and Materials:** A total of 30 IMRT plans from cervical carcinoma patients were retrospectively analyzed on the Trilogy and Eclipse v13.5 treatment planning systems (TPSs). For each patient, a new plan based on Halcyon was created with the same planning parameters and optimization constraints using the Eclipse Version 15.1 TPS. To compare plan qualities, dosimetry parameters regarding planning target volume (PTV), organs at risk (OARs), monitor unit (MU) efficiency, segment size and treatment time were evaluated. Evaluation of the helical diode array system was performed with gamma-index analysis.

**Results:** The dose distribution of the target volume of the Halcyon and Trilogy plans showed no significant difference (*p* > 0.05). The mean doses of rectum and both femoral heads for Halcyon plans were significantly reduced compared to those for Trilogy plans (*p* < 0.05). Compared to Trilogy, Halcyon increased the number of MUs from 1542.9±248.3 MU to 2514.9±328.2 MU (*p* = 0.00) and decreased the delivery time from 11.28±1.36 min to 3.26±0.26 min (*p* = 0.00). The average segment areas of Halcyon plans for proximal and distal multileaf collimators (MLCs) were 42.1 ± 31.2 cm^2^ and 28.4 ± 23.7 cm^2^, respectively, and that of Trilogy plans was 27.3 ± 16.9 cm^2^. The mean gamma index (3 mm/3%) results for the Halcyon and Trilogy plans were 99.41±0.26 and 99.76±0.32 (*p* > 0.05), respectively.

**Conclusions:** All Halcyon treatment plans were recognized as clinically acceptable and had statistically better OAR sparing with higher delivery efficiency. The Halcyon system exhibited fast treatment delivery of IMRT with good dosimetric agreement using ArcCHECK.

## Introduction

In recent years, with the continuous development of precision radiotherapy, intensity-modulated radiotherapy (IMRT) and volumetric-modulated radiotherapy (VMAT) technologies have been widely used 1,2,3. The new treatment technologies give the tumor target enough of a dose with better protection of normal tissues in cervical carcinoma 4,5. Because of the inherent complexities, VMAT is more demanding in terms of quality assurance (QA) than conventional IMRT. Moreover, IMRT is always faster to optimize, and a carefully selected beam angle may provide better dosimetric results than VMAT. Many efforts have been devoted to reducing the treatment times in IMRT 6,7,8.

For accurate IMRT delivery, it has been suggested that simple adjustments to multileaf collimator (MLC) segmentation could allow the photon fluence of the beam to be entirely modulated without any need for a flattening filter (FF). Studies have pointed out that flattening filter free (FFF) beams have a significant impact on the therapeutic efficiency, comfort, imaging quality, dose calculation, radiation protection, etc. 6,7. Therefore, increasingly more attention has been paid to this issue in recent years 9.

The MLC is a critical component to provide an efficient means of shaping the treatment field 10. MLC properties with potential clinical effects, including the leaf width 11,12, transmission 13,14 and speed 14, have been extensively investigated. Lower MLC transmission may have advantages in organ at risk (OAR) sparing 13, and MLCs with very fast motion have been shown to reduce IMRT/VMAT delivery times 5. This may be more obvious for IMRT.

The novel Halcyon (Varian Medical Systems, Palo Alto, CA, USA) accelerator uses FFF mode beams (800 MU/min) and is configured with an alternative design for a dual-layer MLC (5 cm/s). The FFF beam and low transmission of MLC may decrease the OAR dose. However, the patient setup of every fraction on Halcyon is verified entirely through megavoltage (MV) imaging, so daily imaging is imposed by the treatment planning system (TPS), and the imaging dose is automatically included in the plan optimization, which may increase the OAR dose. In addition, use of an FFF beam and high leaf speed with fast gantry rotation (15 sec/rotation) may lead to high delivery efficiency. Studies of the clinical applicability of Halcyon are therefore of interest. This study analyzes different plan qualities and delivery efficiencies for the Halcyon and Trilogy accelerators.

## Methods and Materials

### Patients and linear accelerators

A total of 30 cervical carcinoma patients who received IMRT using Trilogy (Varian Medical Systems, Palo Alto, CA, USA) were randomly enrolled in this study. The clinical target volume (CTV) of each patient was contoured to include the primary tumor area, uterus, and pelvic and para-aortic lymph nodes. The corresponding planning target volume (PTV) was generated by symmetrically expanding 0.5 cm from the CTV. The mean ± standard deviation (SD) of the PTV volumes was 838.8±152.3 cc.

The Trilogy features a 6 MV FF mode. The beam-shaping collimation consists of upper and lower jaws with a static jaw technique followed by an MLC system. The Trilogy was equipped with a Millennium MLC with a 14.5 cm limit on overtravel of the leaves. The Millennium MLC has 120 leaves, with a maximum leaf speed of 2.5 cm/sec. The central 80 leaves and the outer 40 leaves have projection leaf widths of 0.5 cm and 1.0 cm, respectively, at the isocenter. The leaf has an 8.0 cm radius of curvature with 6.5 cm height. The Halcyon (Varian Medical Systems, Palo Alto, CA, USA) accelerator is capable of delivering an FFF 6 MV beam with a maximum dose rate 800 MU/min. The device is configured with a dual-layer MLC (SX1 mode). The proximal bank pairs have 58 leaves, and the distal bank pairs have 56 leaves with a standard width of 1 cm at the isocenter. The maximum moving speed of leaves on both layers is 5 cm/sec relative to the isocenter. The leaf transmission was reduced by stacked and staggered leaves. Each leaf can reach across the entire 28 cm field and has a 23.4 cm radius of curvature with 7.7 cm height.

### Treatment planning

Each patient was delivered a daily fraction of 1.8 Gy up to the total prescription dose of 50.4 Gy in 28 fractions. All plans were normalized to cover 95% of the PTV by the prescription dose. The volume of the bladder and rectum receiving 40 Gy (V_40_) should not exceed 35%. The restriction for the femoral head was V_40_<5%. To reduce the dosage for the normal tissue as much as possible, we adjusted the dose-volume optimization constraints to meet the target dose coverage and spare more OARs.

The original IMRT plans were retrospectively analyzed and designed using the Varian Eclipse V13.5 treatment planning system (TPS for the Trilogy accelerator. The Varian Trilogy accelerator used 6 MV photon beams and operated in an FF mode. The fixed field IMRT plan used 7 field angles (0˚, 51˚, 102˚, 153˚, 204˚, 255˚ and 310˚). The optimization and dose calculation in Eclipse used the Photon Optimizer (PO, version 13.5.35) and anisotropic analytical algorithm (AAA, version 13.5.35) with a grid resolution of 2.5×2.5×2.5 mm^3^. Thirty planning cases, as mentioned above, were imported into Eclipse V15.1.15 TPS using the Halcyon accelerator in the SX1 mode of MLC operation to redesign plans in the 6 MV FFF mode with the same parameters and constraints, as mentioned above. The version of PO and AAA was 15.1.15 for the Halcyon plans.

### Plan evaluation

The dose-volume histograms (DVHs) of the target and OARs and the RTplan files were exported from the two versions of the planning system. Using an in-house-developed MATLAB 2010b software package, the dosimetric information and segment area were analyzed from the DVH and RTplan files.

For the dosimetric evaluation, the following indices were calculated: the maximum and minimum doses, represented by the doses received by 2% (D_2%_) and 98% (D_98%_) of the target volume, respectively. Additionally, the homogeneity index (HI), conformity index (CI) and gradient index (GI) of the target were calculated. They were as follows:
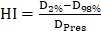
, 
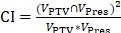
 and 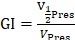, where V
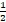
_Pres_ is the volume of the 50% prescription dose line. For OARs, the mean dose (D_mean_) and the volumes of the bladder, rectum and femoral heads receiving more than 20, 30 and 40 Gy (V_20_, V_30_ and V_40_) were reported. The volume of normal tissue receiving >5 Gy was recorded for the body outline excluding the PTV.

### Delivery verification

The total number of monitor units (MUs) and the segment areas from the Trilogy and Halcyon were compared. Patient-specific quality assurance was performed on an ArcCHECK helical dosimetry diode array, and the delivery accuracy was evaluated via gamma-index analysis (3%/3, 3%/2, 2%/3, 2%/2-mm) with a 10% lower dose exclusion threshold.

### Statistical analysis

The statistical significance of any difference between the Trilogy plan values and Halcyon optimization wFas assessed by the Wilcoxon signed-rank test, where a p-value <0.05 was considered statistically significant.

## Result

### PTV coverage

**Figure 1:**
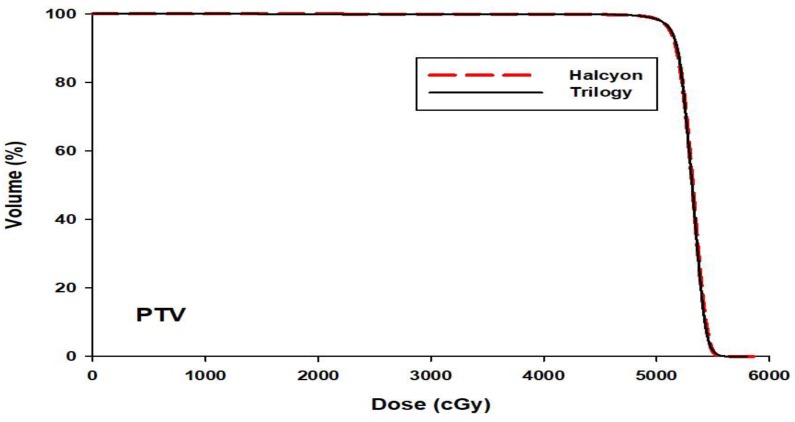
The mean DVHs of PTV averaged over 30 patients for the Trilogy (black solid) and Halcyon (red dashed) plans

The mean DVHs of the target for 30 patients are shown in Fig. 1. The statistical results are reported in Table 1. There was no significant difference for D_2%_, D_98%_, HI, CI and GI between plans generated using Halcyon and Trilogy (*p* > 0.05). The D2 and D98 in the Halcyon IMRT plans were slightly larger than those of the Trilogy IMRT program, which were 54.86 ± 0.16 vs 54.74± 0.42 (*p* = 0.19) and 50.35 ± 0.42 vs 50.25 ± 0.59 (*p* = 0.57), respectively. CI and GI were slightly lower for Halcyon plans than Trilogy plans, but neither result was statistically significant (*p* > 0.05).

### OARs

**Figure 2:**
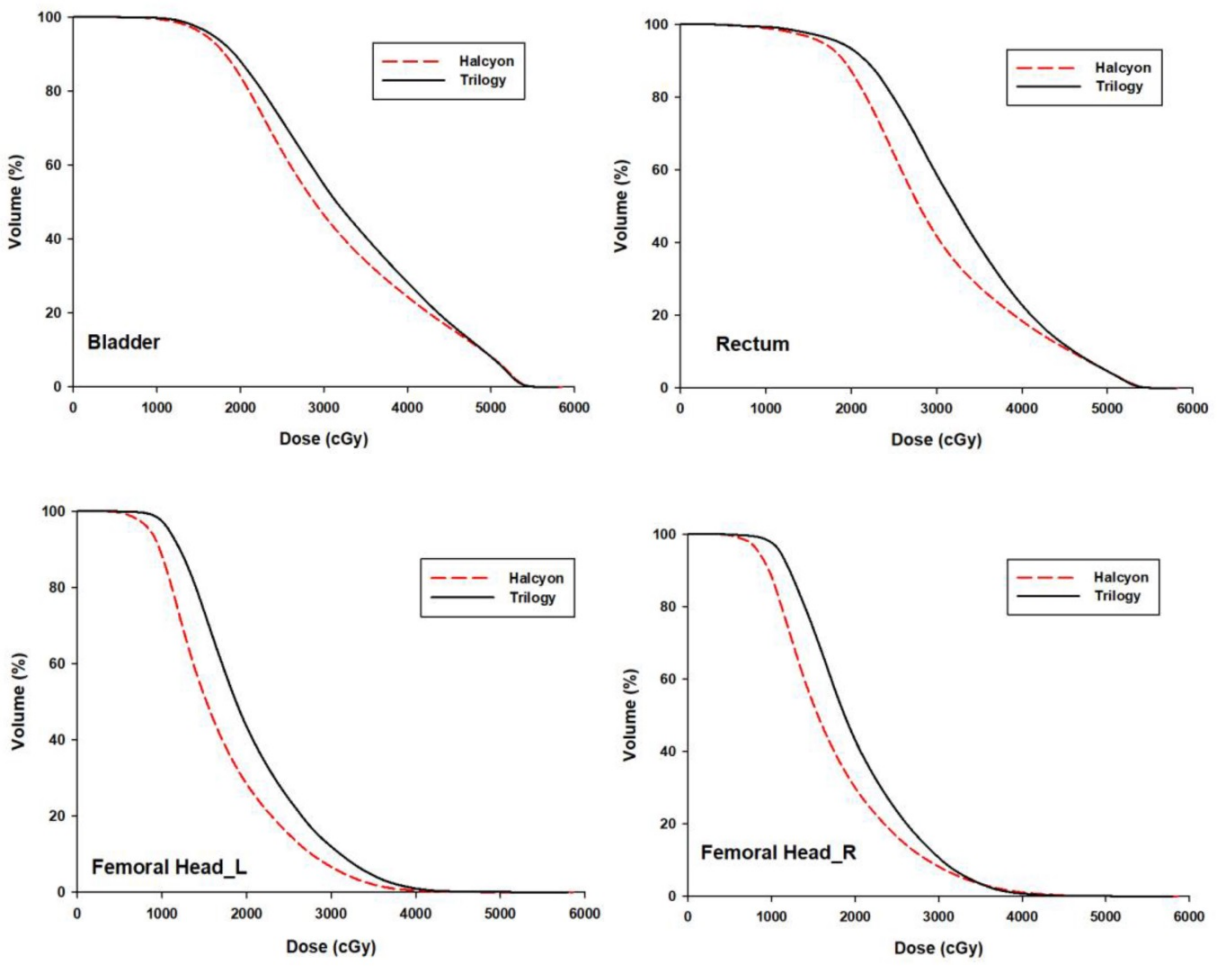
The mean DVHs of OARs averaged over 30 patients for the Trilogy (black solid) and Halcyon (red dashed) plans.

Average DVHs for the bladder, rectum and femoral heads are shown in Fig. 2. The dosimetric statistical results are summarized in Table 1. V_30_ of the bladder and rectum and, V_20_ of both femoral heads were significantly lower in Halcyon plans than in Trilogy plans (*p* <0.05). The mean dose of OARs for both femoral heads and rectum was significantly lower in Halcyon plans (*p* <0.05). The mean dose for the rectum, left femoral head and right femoral head was reduced by 2.65 Gy, 3.27 Gy, and 2.65 Gy, respectively. The D_mean_ and V_5_ of the normal tissue for Halcyon were less than those for the Trilogy plans. However, these differences were not statistically significant (*p* > 0.05).

### Delivery efficiency

**Figure 3:**
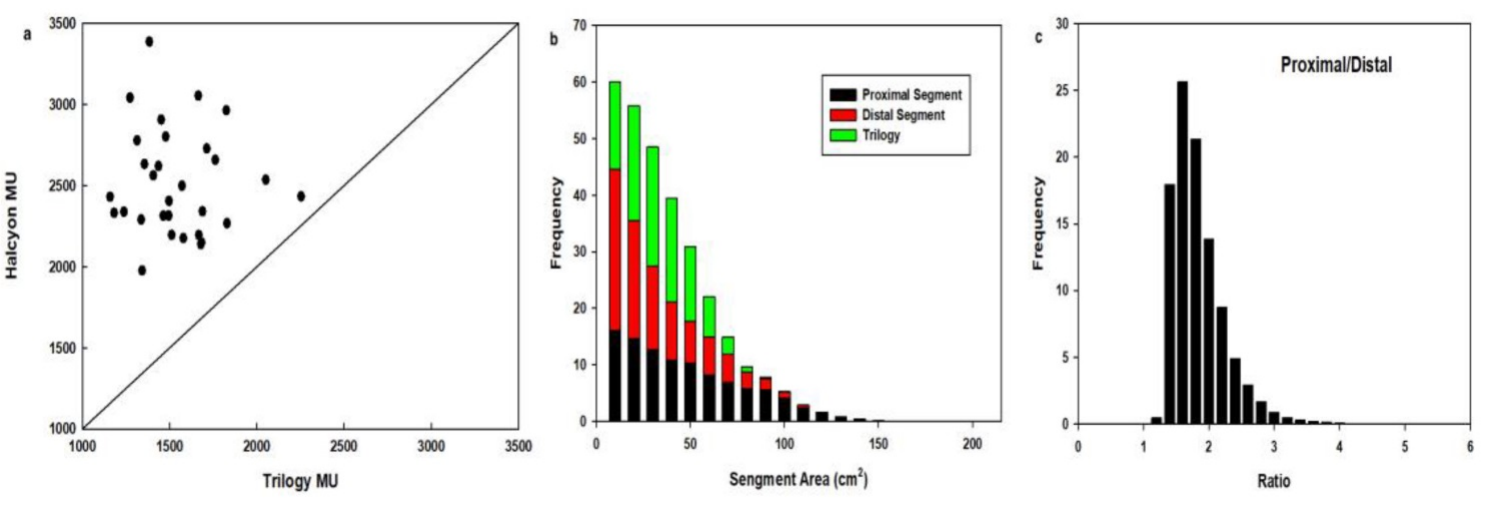
Comparison of MUs (a), segment areas (b) and the segment area ratio of proximal MLC to distal MLC (c) for 30 patients.

Fig. 3. shows that compared to the number of MUs of Trilogy, those of Halcyon were increased (1542.9±248.3 vs. 2514.9±328.2, *p* = 0.00), whereas the delivery time was decreased from 11.28±1.36 min to 3.26±0.26 min (*p* = 0.00). The average area of the proximal segment of Halcyon was 42.1 ± 31.2 cm^2^, and that of the distal segment was 28.4 ± 23.7 cm^2^, whereas the average segment area of Trilogy was 27.3 ± 16.9 cm^2^. The area ratio between proximal and distal segments was 1.74 ± 0.43, ranging from 0.42 to 8.48, and the percentage of proximal segment area that was greater than the distal segment was 0.046%.

### Verification comparison

Table 2 indicates that with the 3%/3 mm criteria, the average passing rates were 99.41%±0.26% and 99.76%±0.32% for the Halcyon and Trilogy plans (*p* > 0.05), respectively, whereas with the 2%/2 mm criteria, the average passing rates were 94.96±1.22 and 95.14±1.89 for the Halcyon and Trilogy plans (*p* > 0.05), respectively.

## Discussion

For patients with cervical carcinoma, IMRT plays a key role in both definitive and adjuvant treatment and has been demonstrated to reduce radiation-induced complications compared to 3D conformal radiotherapy (3D-CRT) 4,15. This study has found that the equivalent target coverage and superior sparing of OARs were achieved with the Varian Halcyon system compared to the original optimized clinically accepted IMRT plans for Trilogy. In addition, it was able to successfully handle the geometric and dosimetric variations in cervical carcinoma for Halcyon with high delivery quality and efficiency.

The Halcyon accelerator uses daily MV imaging for patient setup, which would introduce additional normal tissue doses. In this study, MV-CBCT images are acquired via a continuous gantry rotation from 260˚ to 100˚, with a total of 10 MUs for the high-quality mode. For MV-CBCT, Li et al. 16. showed that the cumulative imaging and treatment plan dose distribution can be expected to accurately reflect the actual dose. Since kilo-voltage imaging offers a lower patient dose and better soft-tissue contrast, KV-CBCT will become available for Halcyon in the near future. However, Halcyon introduces a number of novel components different from Trilogy that could uniquely impact plan quality. FFF capabilities, no backup jaws and dual MLC leaves are included. In this study, for 30 cervical carcinoma IMRT plans, the target area and the OAR exposure dose can meet the clinical requirements. There were no significant differences in D_2%_, D_98%_, CI, HI and GI between the Halcyon and Trilogy platforms. Despite the superposition of the dose of MV-CBCT, Halcyon plans outperform the Trilogy plans in reducing normal organ doses. This difference may be attributed to the low transmission two-layer MLC and the FFF beam.

Compared to 3D-CRT, IMRT can achieve better conformal dose distributions to concave tumor shape and spare nearby normal tissues using field fluence optimization. The beam intensity in the field can be modulated as needed using MLC 1,2,3. There are many design characteristics in each MLC, such as the leaf numbers, leaf width and leakage, to name a few 10. Wang et al. 11 demonstrated that the dose coverage of PTV using a 4-mm MLC was superior to that of a 10-mm MLC for prostate IMRT. However, Jacob et al. 12 concluded that the plan quality was equivalent in conformal coverage of the target volume and sparing of organs at risk using the three collimator leaf thicknesses studied (10, 5 and 2.5 mm) in IMRT. There was no uniform agreement regarding the benefit of smaller leaf width. The leaf actual physical width was thinner, and mechanical processing was more difficult. For Halcyon MLC, the width is 1.0 cm for both layer leaves, and the accelerator has full field travel distance with 100% interdigitization capability.

Leaf transmission is an important factor that influences IMRT delivery. The transmission ratio of the Varian millennium120 MLC was measured to be 1.4% and 1.5% in the studies conducted by Li et al. 14 and Yao et al. 17 using different measurement methods. To reduce the transmission and leakage between the leaves, a collimator jaw is usually used in conventional accelerators. To investigate the jaws' effect on the dose distribution, some studies have been performed. Joy et al. analyzed the dose effects of jaw tracking in static IMRT, the results indicated that the volume of the low dose region was reduced and the maximum reduction in V_5_ for the normal tissue was 16.7% 18. Schmidhalter et al. showed that the undesired dose in OARs was decreased by leaf transmission reduction using moving jaws 19. Jaw tracking keeps only the jaws as close as possible to the MLC aperture during dose delivery, and leaf transmission and leakage were further deceased by the jawless MLC of the Halcyon, which was composed of two staggered layers. In the study of Roover et al. with the Halcyon MLC, the measured single-layer MLC transmission ratio was 0.42% 20. Lim et al. have reported that the measured leaf transmission was 0.41% for distal-only and 0.40% for proximal-only 21. Other multilayer MLC systems were proposed earlier and add a high-resolution field-shaping ability 22,23,24. The MLC transmission is further reduced by using FFF beams 25. Vassiliev et al. analyzed the beam characteristics of the Varian 21EX accelerator with and without an FF and showed that the FFF beam field dose decreased, the total scattering factor decreased with the change in the field, and the change decreaFfigsed in the lateral dose curve with depth 26. These characteristics are conducive to the protection of normal tissues.

Treatment time reductions have a potential clinical impact in terms of reducing the risk of intrafraction motion and enhancing patient comfort during treatment. Treatment times depend on the time required to irradiate the MUs on one hand and on the time for gantry and MLC movement on the other hand. Recently, the Agility MLC (Elekta, Crawley, UK) with 160 leaves with very fast motion instead of MLCi has been shown to reduce 6.5 min/42.5% for IMRT with 9 beams 5. In the current study, the delivery time for Halcyon was 3.26±0.26 min, which was improved by approximately 8 min over that of Trilogy. The Halcyon system allows a maximum gantry rotation speed of 4RPM for IMRT and 2 RPM VMAT delivery. The two arc VMAT plan delivery time on Halcyon for head and neck cancer has been cut by nearly half compared to C-arm linac 27. In our experience, 7 fields IMRT has a delivery time similar to that of 2 arc VMAT for cervical carcinoma.

Small irregularly shaped fields are common in IMRT plans, of which the dosimetry has been one of the largest challenges, i.e., smaller fields require detailed knowledge of output factors and careful dosimetry 28. Distributions and selections of larger segment areas generally lead to higher MU efficiencies 29,30. In SX1 mode, the lower layer of leaves is used for field shaping; the upper leaf is there only to perform leaf tracking and does not contribute to the modulation itself. The segment areas of lower leaves in Halcyon plans were relatively larger than those in Trilogy, which means that the delivery time is shorter and that the MU efficiency is higher in IMRT. However, the maximum field size of Halcyon was limited to 28×28 cm^2^, which means that it is not suitable for too-large tumors in one isocenter plan. In addition, when looking at deep-seated tumors, the use of the higher-energy photon beam of Trilogy may be advantageous since Halcyon offers only a 6-MV photon beam.

Furthermore, the corresponding segment ratio of the upper and lower leaves was analyzed. More than 99.95% of the area ratio is greater than 1, indicating that the segment area of the lower leaves is less than the upper leaves when the plan is optimized. Field area ratios can be up to 8.48. There is also a small amount of the upper segment area that is smaller than the lower area in Halcyon IMRT plan in cervical cancer. This shows that fine QA is expected in the upper and lower layers of MLC dose and mechanical parts. It has been widely reported that the MLC leaf root mean square (RMS) error was closely linked to maximum leaf speeds 31,32,33,34. The pretreatment QA of ArcCHECK has shown that Halcyon accurately delivers the calculated dose distribution. Further investigation to determine the position error and actual leaf speeds of this MLC during IMRT and VMAT is underway.

Utilizing FFF mode, fast leaf velocity delivery and larger segment areas yielded the greatest improvement in treatment time. FFF mode can reduce the OAR dose and increase dose rate. The new MLC has lower leaf transmission and a higher motion speed, which will have better OAR sparing and reduction in delivery time. A main drawback of the current study is that it is not possible to distinguish the exact contribution of each of these factors.

In conclusion, the Varian Halcyon system may produce fixed-field IMRT plans. Overall, it was as comparably sparing as the original clinically acceptable plans from Trilogy in the pelvic region, with high efficiency and good dosimetric agreement.

## Figures and Tables

**Table 1:** Target coverage metrics and OAR doses

Structures		Parameters	Halcyon	Trilogy	*z*	*p*
PTV		D_2%_(Gy)	54.86±0.16	54.73±0.34	-2.36	0.190
		D_98%_(Gy)	50.35±0.42	50.25±0.59	-0.57	0.573
		HI	0.09±0.01	0.09±0.02	-0.47	0.645
		CI	0.81±0.04	0.83±0.03	-3.05	0.206
		GI	5.05±0.84	5.06±0.79	-0.29	0.773
OAR	Bladder	V_20_(%)	83.14±8.54	88.01±7.21	-0.23	0.822
		V_30_(%)	46.25±9.10	54.87±8.95	-2.20	**0.028**
		V_40_(%)	24.37±6.87	28.41±7.62	-3.34	**0.001**
		D_mean_(Gy)	31.04±2.57	32.83±2.26	-1.27	0.081
	Rectum	V_20_(%)	86.72±11.32	92.91±5.58	-2.02	**0.044**
		V_30_(%)	42.35±12.38	58.26±9.76	-4.47	**0.001**
		V_40_(%)	19.05±8.62	22.94±9.40	-1.46	0.146
		D_mean_(Gy)	30.13±3.13	32.78±2.30	-3.23	**0.001**
	Femoral Head_L	V_20_(%)	29.00±9.49	44.30±13.20	-4.43	**0.000**
		V_30_(%)	6.81±3.84	12.21±8.71	-2.84	**0.005**
		V_40_(%)	0.41±0.89	0.96±1.62	-2.27	**0.023**
		D_mean_(Gy)	17.18±2.20	20.45±2.46	-4.53	**0.000**
	Femoral Head_R	V_20_(%)	30.30±9.13	43.95±11.40	-4.44	**0.000**
		V_30_(%)	8.39±4.5	10.91±9.12	0.56	0.575
		V_40_(%)	1.11±1.41	0.71±1.72	-2.08	0. 370
		D_mean_(Gy)	17.57±2.24	20.22±2.16	-3.91	**0.000**
	Normal Tissue	V_5_(%) D_mean_(Gy)	51.24±8.63 11.58±1.86	51.86±7.78 11.53±1.67	-0.699 -0.118	0.440 0.85

**Table 2:** Delivery accuracy by average gamma evaluation passing rate of the ArcCHECK measurements

Passing rates	Halcyon	Trilogy	z	p
3%/3 mm	99.41±0.26	99.76±0.32	-2.45.	0.362
3%/2 mm	98.14±0.72	97.97±0.88	-2.28	0.731
2%/3 mm	98.00±0.75	98.37±0.64	-1.84	0.205
2%/2 mm	94.96±1.22	95.14±1.89	-1.19	0.811
